# Total and High Molecular Weight Adiponectin and Hepatocellular Carcinoma with HCV Infection

**DOI:** 10.1371/journal.pone.0026840

**Published:** 2011-11-14

**Authors:** Shuji Sumie, Takumi Kawaguchi, Ryoko Kuromatsu, Akio Takata, Masahito Nakano, Manabu Satani, Shingo Yamada, Takashi Niizeki, Takuji Torimura, Michio Sata

**Affiliations:** 1 Division of Gastroenterology, Department of Medicine, Kurume University School of Medicine, Kurume, Japan; 2 Department of Digestive Disease Information and Research, Kurume University School of Medicine, Kurume, Japan; 3 Liver Cancer Research Division, Research Center for Innovative Cancer Therapy, Kurume University, Kurume, Japan; National Cancer Institute, United States of America

## Abstract

**Background:**

Adiponectin is shown to be inversely associated with development and progression of various cancers. We evaluated whether adiponectin level was associated with the prevalence and histological grade of hepatocellular carcinoma (HCC), and liver fibrosis in patients with hepatitis C virus (HCV) infection.

**Methods:**

A case-control study was conducted on 97 HCC patients (cases) and 97 patients (controls) matched for sex, Child-Pugh grade and platelet count in patients with HCV infection. The serum total and high molecular weight (HMW) adiponectin levels were measured by enzyme-linked immunosorbent assays and examined in their association with the prevalence of HCC. In addition, the relationship between these adiponectin levels and body mass index (BMI), progression of liver fibrosis, and histological grade of HCC was also evaluated. Liver fibrosis was assessed using the aspartate aminotransferase to platelet ratio index (APRI).

**Results:**

There were no significant differences in the serum total and HMW adiponectin levels between cases and controls. Moreover, there were no inverse associations between serum total and HMW adiponectin levels and BMI in both cases and controls. On the other hand, serum total and HMW adiponectin levels are positively correlated with APRI in both cases (r = 0.491, P<0.001 and r = 0.485, P<0.001, respectively) and controls (r = 0.482, P<0.001 and r = 0.476, P<0.001, respectively). Interestingly, lower serum total (OR 11.76, 95% CI: 2.97–46.66 [P<0.001]) and HMW (OR 10.24, CI: 2.80–37.40 [P<0.001] adiponectin levels were independent risk factors of worse histological grade of HCC.

**Conclusions:**

Our results suggested that serum total and HMW adiponectin levels were predictors of liver fibrosis, but not prevalence of HCC in patients with HCV infection. Moreover, low these adiponectin levels were significantly associated with worse histological grades.

## Introduction

Hepatocellular carcinoma (HCC) is one of the most common malignancies in the world. The incidence of HCC has increased in Eastern Asia and Africa during the past several decades, and has also increased in the United States [1]. In many countries, this trend is attributed to hepatitis C virus (HCV) infections, and in Japan, over 70% of all HCC are related to chronic liver disease with HCV infection [2]. In order to prevent and treat this malignancy, it is important to understand the pathogenesis of HCC in patients with HCV infection.

Obesity is widely recognized as a significant risk factor for the development of various cancers [3]. It also is suggested that obesity is associated with the progression of chronic liver disease [4], [5] and with HCC [6]. Especially, nonalcoholic fatty liver disease is recognized to be a hepatic manifestation of the metabolic syndrome and obesity and insulin resistance play a major role in the pathogenesis [7]. Nonalcoholic fatty liver disease represents a spectrum of conditions that are histologically characterized from simple steatosis to nonalcoholic steatohepatits, which is associated with increased risk of advanced liver fibrosis, cirrhosis and development of HCC [8]. Similarly, in patients with HCV infection, several studies have shown that obesity was associated with disease progression [4], [5] and with HCC development [9], [10]. However, it is unclear how obesity is linked to development of HCC in patients with HCV infection.

Adiponectin is a peptide hormone secreted by adipocyte and hepatocyte [11]. Adiponectin exists primarily in three forms: low molecular weight trimers, medium molecular weight hexamers, and high molecular weight (HMW) multimers [12]. Among three forms, HMW adiponectin is thought to have more biological activity than other forms of adiponectin [12], [13]. Adiponectin has antiatherogenic, antiinflamatory and insulin-sensitizing actions and is inversely associated with body mass index (BMI); therefore it is also linked to the various metabolic abnormalities associated with obesity [14]. Recently, hypoadiponectinemia has been shown to be an important risk factor for the development of various cancers associated with obesity such as breast cancer [15], endometrial cancer [16], colorectal cancer [17], and gastric cancer [18]. However, it is still unknown whether adiponectin contribute to the development of HCC in patients with HCV infection. Moreover, adiponectin has been shown to be associated with progression of liver fibrosis in patients with chronic liver disease [19], [20], [21]. However, in patients with HCV infection, the association between adiponectin and liver fibrosis is uncertain.

Histological grade is known to be one of the most important risk factor for patients with HCC. Several studies have shown that histological grade affected recurrence and survival after curative resection and liver transplantation for HCC [22], [23]. Although it is known that worse histological grade is associated with high cell proliferation and angiogenesis of HCC, these molecular mechanisms is not unclear. Several clinical studies have demonstrated that serum adiponectin levels were negatively associated with increasing of histological grades of several cancers [15], [24], [25].

Therefore, in case-control study, we investigated the association of serum total and HMW adiponectin levels with prevalence of HCC in chronic liver disease with HCV infections. In addition, the associations between these adiponectin levels and BMI, progression of liver fibrosis, and histological grades of HCC were also examined.

## Materials and Methods

### Ethics Statement

The study protocol was approved by The Ethical Committee of Kurume University, and written informed consent for participation in the study was obtained from each subject and conformed to the guidelines of the 1995 Declaration of Helsinki.

### Patients

Between January 1997 and December 2007, 97 Japanese cases with chronic HCV infection at the Kurume University School of Medicine were diagnosed with HCC and enrolled in this study. All cases had no medical history of previous or present neoplastic disease at any other site. The case patients were histologically confirmed with HCC by needle biopsy, and with a single tumor ≤5 cm or three or fewer tumors each ≤3 cm seen on ultrasonography and computed tomography. Histological classification was based on Liver Cancer Study Group of Japan [26]. According to Edmondson-Steiner classification [27], well differentiated corresponds to grade I and a part of grade II, and moderately differentiated corresponds to grade II and grade III with a clear trabecular pattern, and poorly differentiated corresponds to grade III with an indistinct trabecular pattern and part of grade IV. Tumor sizes were determined based on the largest dimension of the tumor. Between January 2005 and December 2007, 97 patients with chronic HCV infection, who matched for sex, Child-Pugh grade and platelet count (±20×10^9^/L), were randomly selected as controls at the same hospital. The control patients had no medical history of previous or present neoplastic disease, including HCC. In all cases and controls, hepatic functional reserve was determined using the Child-Pugh scoring system. Diabetes was defined as a fasting blood glucose ≥126 mg/dl, and/or a random blood glucose ≥200 mg/dl. BMI was calculated as body weight in kg divided by the square of the height in meters (kg/m^2^).

### Assessment of liver fibrosis

Liver fibrosis was assessed using the aspartate aminotransferase (AST) to platelet ratio index (APRI) in this study. APRI has been recognized as a noninvasive test to characterize the degree of liver fibrosis in chronic liver disease with HCV infection [28]. APRI was calculated for all study subjects as follows: AST/upper limit of normal (45 IU/L)×100/platelet count (10^9^/L).

### Markers of hepatic virus

HCV infection was evaluated using anti-HCV antibody (HCV-Ab). The diagnosis of HBV infection was also based on detection of hepatitis B surface antigen (HBsAg). The presence of HCV-Ab and HBsAg was determined using standard clinical methods (Department of Clinical Laboratory, Kurume University Hospital). All cases and controls were positive for HCV-Ab and negative for HBsAg in this study.

### Measurement of serum adiponectin

Fasting morning blood samples were obtained from all subjects and stored at −20°C for later analysis. Blood samples were collected by all cases before HCC therapy was initiated. Serum total and HMW adiponectin levels were measured by enzyme-linked immunosorbent assays using the Human Adiponectin Latex Kit (Eiken Chemical Co., Ltd., Tokyo, Japan) and High Molecular Weight Adiponectin Assay Kit (Fujirebio Inc., Tokyo, Japan), respectively.

### Statistical Analysis

Continuous variables were expressed as mean ± standard deviation. Comparisons between the 2 groups were performed using the Mann-Whitney U test for continuous variables, and the chi-square test for discrete variables. Pearson correlation test was used to evaluate the association between plasma total and HMW adiponectin levels and BMI and APRI in each cases and controls. Comparison analysis between histological grades was performed by the one-way ANOVA with Bonferroni corrections for post hoc comparisons. The relationships between total and HMW adiponectin and HCC histological grades of were determined using multiple logistic regression models. Data were reported as odds ratios (ORs) and 95% confidence intervals (95% CIs). All *P* values were 2-tailed, and *P*<0.05 was considered to be statistically significant. Statistical analysis was performed using SPSS software (SPSS Inc., Chicago, IL).

## Results

### Serum adiponectin levels and prevalence of HCC

**Figure 1:**
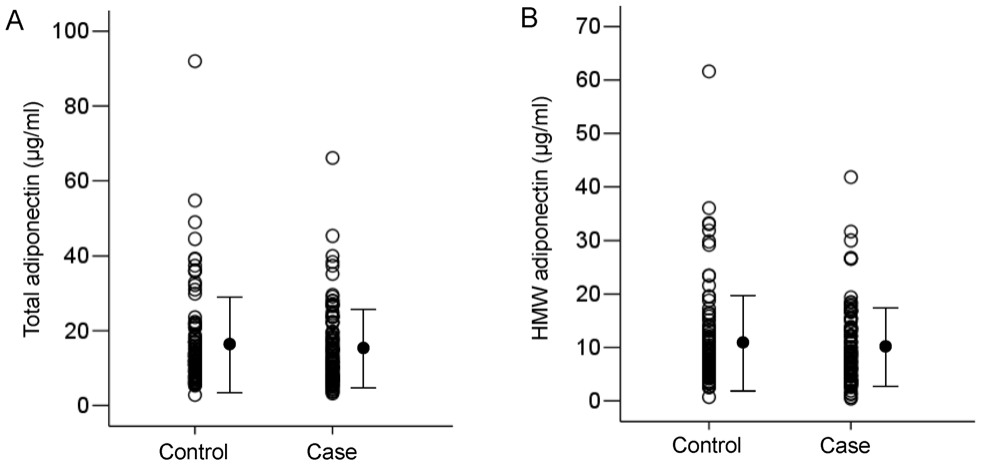
Comparison of adiponectin levels between 97 controls and 97 cases. A. Comparison of total adiponectin levels between patients with cases and controls (*P* = 0.670). B. Comparison of HMW adiponectin levels between patients with cases and controls (*P* = 0.752).

The baseline clinical characteristics of the 97 cases and 97 controls were shown in Table 1. No significant differences were found between cases and controls according to AST level, alanine transaminase (ALT) level, APRI, diabetes mellitus, or BMI. The mean age of the cases was significantly higher than the mean age of the controls. The associations between serum total and HMW adiponectin levels and prevalence of HCC were shown in Figure 1. There were no significant differences between the mean total adiponectin levels of the cases and controls (15.5±10.4 µg/ml and 16.6±12.8 µg/ml, respectively, *P* = 0.670) (Figure 1A), or the mean HMW adiponectin levels (10.1±7.4 µg/ml and 10.8±9.0 µg/ml, respectively, *P* = 0.752) (Figure 1B).

**Table 1:** Baseline clinical characteristics in case and control.

	Case	Control	P value
**Number**	97	97	
**Age (years)**	67.4±8.3	61.2±11.4	<0.001
**Gender (Female/Male)**	30/67	30/67	Matched variable
**AST (U/L)**	61.8±28.3	60.0±28.5	0.632
**ALT (U/L)**	58.4±33.4	60.0±31.8	0.513
**Platelet (×10^9^/L)**	115.0±51.4	115.5±52.5	Matched variable
**APRI**	1.5±1.0	1.5±1.1	0.863
**Child-Pugh grade (A/B+C)**	82/15	82/15	Matched variable
**Diabetes mellitus (Absent/Present)**	64/33	71/26	0.275
**BMI (kg/m^2^)**	22.5±3.2	23.1±3.3	0.228
**Total adiponectin (µg/ml)**	15.5±10.4	16.6±12.8	0.670
**HMW adiponectin (µg/ml)**	10.1±7.4	10.8±9.0	0.752

Continuous variables presented as mean ± standard deviation.

Abbreviation: AST = aspartate aminotransferase; ALT = alanine aminotransferase;

APRI = aspartate aminotransferase-to-platelet ratio index; BMI = body mass index;

HMW = high molecular weigh.

### Association between serum adiponectin levels and BMI

**Figure 2:**
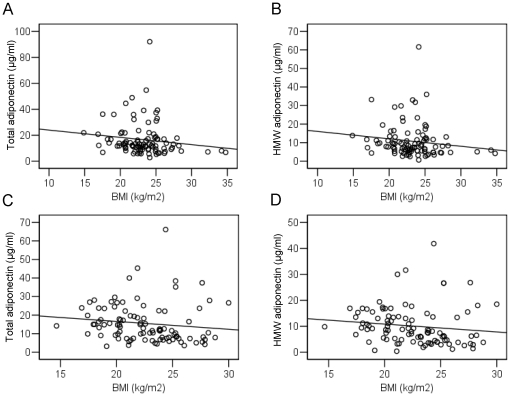
Serum adiponectin levels and body mass index (BMI). A. Correlation between serum total adiponectin levels and BMI in controls (r = −0.142, *P* = 0.166). B. Correlation between serum high molecular adiponectin (HMW) adiponectin levels and BMI in controls (r = −0.144, *P* = 0.160). C. Correlation between serum total adiponectin levels and BMI in cases (r = −0.129, *P* = 0.208). D. Correlation between serum HMW adiponectin levels and BMI in cases (r = −0.131, *P* = 0.201).

We examined the associations between serum total and HMW adiponectin levels and BMI in cases and controls. In controls, there were no inverse associations between serum total and HMW adiponectin levels and BMI (r = −0.142, *P* = 0.166 for total adiponectin [Figure 2A] and r = −0.144, *P* = 0.160 for HMW adiponectin [Figure 2B]). Similarly, in cases, there were also no inverse associations between serum total or HMW adiponectin levels and BMI (r = −0.129, *P* = 0.208 for total adiponectin [Figure 2C] and r = −0.131, *P* = 0.201 for HMW adiponectin [Figure 2D]).

### Association between serum adiponectin levels and APRI

**Figure 3:**
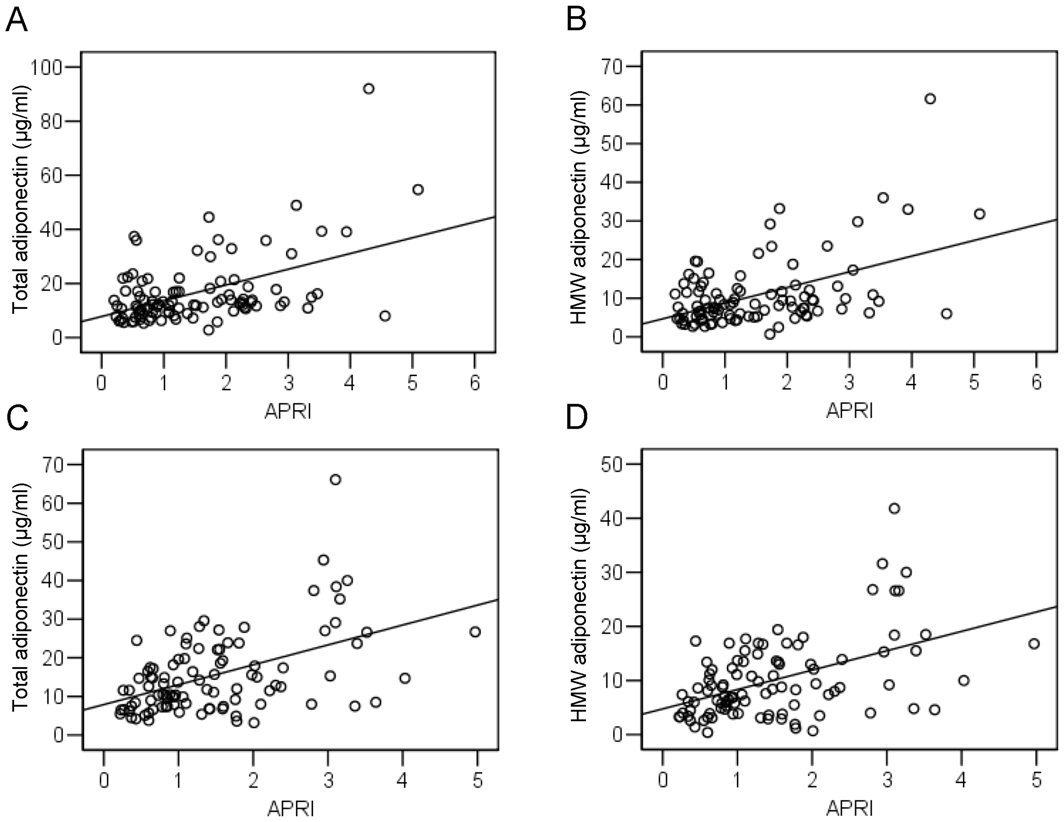
Serum adiponectin levels and aspartate aminotransferase-to-platelet ratio index (APRI). A. Correlation between serum total adiponectin levels and APRI in controls (r = 0.482, *P*<0.001). B. Correlation between serum high molecular adiponectin (HMW) adiponectin levels and APRI in controls (r = 0.476, *P*<0.001). C. Correlation between serum total adiponectin levels and APRI in cases (r = 0.491, *P*<0.001). D. Correlation between serum HMW adiponectin levels and APRI in cases (r = 0.485, *P*<0.001).

We also evaluated the associations between serum total and HMW adiponectin levels and APRI in cases and controls. In controls, serum levels of total and HMW adiponectin were positively associated with APRI (r = 0.482, *P*<0.001 for total adiponectin [Figure 3A] and r = 0.476, *P*<0.001 for HMW adiponectin [Figure 3B]). Similarly, in cases, serum levels of total and HMW adiponectin were positively associated with APRI (r = 0.491, *P*<0.001 for total adiponectin [Figure 3C] and r = 0.485, *P*<0.001 for HMW adiponectin [Figure 3D]).

### Comparison of serum adiponectin levels according to HCC histological grades

**Figure 4:**
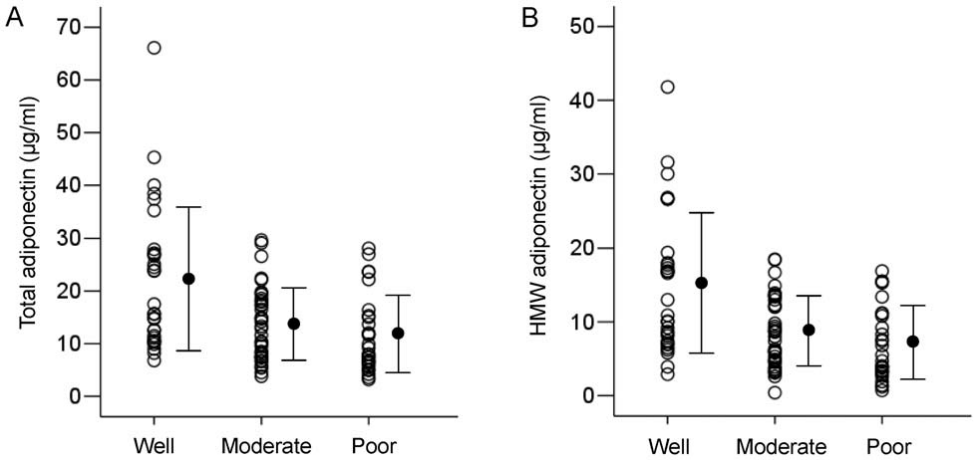
Comparison of adiponectin levels according to histological grades in 97 cases. A. The serum total adiponectin levels in patients with moderately (*P* = 0.001) and poorly (*P*<0.001) differentiated hepatocellular carcinoma (HCC) were significantly lower compared to those in patients with well-differentiated HCC. B. The serum high molecular weight (HMW) adiponectin levels in patients with moderately (*P*<0.001) and poorly (*P*<0.001) differentiated HCC were significantly lower compared to those in patients with well-differentiated HCC.

The baseline clinical characteristics of 97 HCC cases were also separately evaluated according to the histological grades (Table 2). No significant differences were found between HCC histological grades and age, gender, AST level, ALT level, platelet count, APRI, Child-Pugh grade, diabetes mellitus, BMI, alpha-fetoprotein (AFP) level, des-gamma-carboxy prothrombin (DCP) level, and number of tumor. Patients with moderately and poorly differentiated HCC had significantly larger tumor sizes than patients with well-differentiated HCC. The associations between serum total and HMW adiponectin levels and HCC histological grades were shown in Figure 4. The mean total adiponectin levels in patients with moderately (13.4±6.9 µg/ml, *P* = 0.001) and poorly (11.5±7.3 µg/ml, *P*<0.001) differentiated HCC were significantly lower compared to those in patients with well-differentiated HCC (22.0±13.6 µg/ml). The mean HMW adiponectin levels in patients with moderately (8.6±4.7 µg/ml, *P*<0.001) and poorly (6.9±5.0 µg/ml, *P*<0.001) differentiated HCC were significantly lower compared to those in patients with well-differentiated HCC (14.9±9.5 µg/ml). In addition, multiple logistic regression analyses were performed to determine whether the serum total and HMW adiponectin levels were independently associated with HCC histological grades (Table 3). In the analysis adjusted for other variables, lower serum total adiponectin levels (12–24 µg/ml; OR: 9.33, 95% CI: 2.27–38.43 [*P* = 0.002], <12 µg/ml; OR 11.76, 95% CI: 2.97–46.66 [*P*<0.001]) and serum HMW adiponectin levels (7–14 µg/ml; OR: 5.67, 95% CI: 1.66–19.33 [*P* = 0.006], <7 µg/ml; OR 10.24, CI: 2.80–37.40 [*P*<0.001]) were independent risk factors for worse HCC histological grades.

**Table 2:** Association between clinical characteristics and histological grade in patients with hepatocellular carcinoma.

	Well	Moderate	Poor	*P* value
**Number**	30	39	28	
**Age (years)**	67.5±6.9	67.3±8.2	67.1±9.9	0.942
**Gender (Female/Male)**	11/19	12/27	7/21	0.630
**AST (U/L)**	67.2±23.8	59.5±28.8	59.3±25.9	0.409
**ALT (U/L)**	61.0±35.5	60.0±33.1	53.5±32.1	0.647
**Platelet count (×10^9^/L)**	103.1±46.5	116.9±47.6	125.1±54.0	0.228
**APRI**	1.8±1.1	1.3±0.9	1.3±0.9	0.213
**Child-Pugh grade (A/B+C)**	22/8	36/3	24/4	0.095
**Diabetes mellitus (Absent/Present)**	24/6	21/18	19/9	0.073
**BMI (kg/m^2^)**	22.5±3.2	22.4±3.0	22.6±3.5	0.949
**AFP (ng/ml)**	51.3±74.4	165.0±448.4	560.1±1508.8	0.065
**DCP (mAU/ml)**	65.5±139.5	464.2±978.8	276.1±586.4	0.070
**Tumor size (mm)**	21.0±4.9	26.8±10.0†	26.6±11.8†	0.027
**Tumor number (single/2–3)**	19/11	29/10	19/9	0.609
**Total adiponectin (µg/ml)**	22.0±13.6	13.4±6.9†	11.5±7.3†	<0.001
**HMW adiponectin (µg/ml)**	14.9±9.5	8.6±4.7†	6.9±5.0†	<0.001

Continuous variables presented as mean ± standard deviation.

Abbreviation: AST = aspartate aminotransferase; ALT = alanine aminotransferase;

APRI = aspartate aminotransferase-to-platelet ratio index; BMI = body mass index;

AFP = alpha-fetoprotein; DCP = des-gamma-carboxy prothrombin; HMW = high molecular weigh.

† P<0.05 for Bonferroni corrected post hoc comparison with well-differentiated hepatocellular carcinoma.

**Table 3:** Association between total and HMW adiponectin and histological grade in patients with hepatocellular carcinoma by multiple logistic regression analysis.

	Model 1		Model 2	
	OR (95%CI)	*P* value	OR (95%CI)	*P* value
**Total adiponectin (µg/ml)**				
24<	Reference		Reference	
12–24	6.67 (1.83–24.3)	0.004	9.33 (2.27–38.43)	0.002
<12	9.87 (2.76–35.2)	<0.001	11.76 (2.97–46.66)	<0.001
**HMW adiponectin (µg/ml)**				
14<	Reference		Reference	
7–14	4.38 (1.40–13.64)	0.011	5.67 (1.66–19.33)	0.006
<7	9.92 (2.91–33.85)	<0.001	10.24 (2.80–37.40)	<0.001

Abbreviation: OR = odds ratio; 95% CI = confidence interval; HMW = high molecular weigh.

Model 1: adiponectin only.

Model 2: adiponectin and covariates in Table 2.

## Discussion

Obesity is known to be associated with various cancers and hypoadiponectinemia has been also shown to be a risk factor for these obesity-associated cancers [15], [16], [17], [18]. Epidemiological evidence of the association between obesity and HCC is also rapidly increasing including patients with HCV infection. In a community-based cohort study, Chen et al. reported that obesity was an independent risk factor for HCC development in anti-HCV-seropositive subjects [9]. Therefore, we hypothesized that serum adiponectin level may be associated with the prevalence of HCC in patients with HCV infection. However, in this case-control study, serum total and HMW adiponectin levels were not significantly and inversely associated with the prevalence of HCC. Similarly, Nkontchou et al. also demonstrated that serum level of adiponectin is not predictive of HCC development in patients with compensated HCV cirrhosis [29]. In patients with HCV infection, it is well known that metabolic abnormalities such as obesity and diabetes are closely associated with hepatic steatosis [4] and severe fibrosis [5]; as a result, obesity affect as a partial factor of HCC development. On the other hand, several recent reports have shown that hypoadiponectinemia was associated with hepatic steatosis only in limited genotype [30] and association between hypoadiponectinemia and fibrosis progression was not found in patients with HCV infection [31]. In addition, we assessed the association between BMI and serum adiponectin levels in cases and controls. Our results showed that these adiponectin levels were not inversely associated with BMI, suggesting that adiponectin is not a cofactor in the development of HCC associated with obesity in patients with HCV infection.

Recently, several studies have been reported that serum adiponectin level was associated with progression of liver fibrosis in patients with chronic liver disease [19], [20], [21]. We also evaluated the association between serum total and HMW adiponectin levels and progression of liver fibrosis. APRI is a useful noninvasive marker for the prediction of liver fibrosis in chronic liver disease with HCV infection [28] and we assessed the degree of liver fibrosis by APRI in this study. As a result, serum total and HMW adiponectin levels were positively and significantly associated with APRI in both cases and controls. Tietge et al. reported that circulating adiponectin level increased in patients with liver cirrhosis, because of reduced liver function as a major source of adiponectin extraction and altered hepatic hemodynamics [20]. Taken together serum total and HMW adiponectin levels may be a predictors of liver fibrosis in patients with HCV infection.

Next, we examined the relationship between serum adiponectin levels and HCC histological grades. Interestingly, low total and HMW adiponectin levels were independent risk factors for worse HCC histological grades. It is generally known that majority of HCC arises as very well-differentiated cancers and proliferate in a stepwise process of dedifferentiation. When small HCC of the early-stage reach around 1.5–2.0 cm, moderately or poorly differentiated cancer tissues develops within the well-differentiated cancer tissue, and well-differentiated cancer tissue are replaced by less differentiated cancer tissue in so-called advanced HCC [32]. In this study, the mean tumor size of well-differentiated HCC was 14.2±3.2 mm, which was significantly smaller than the mean size of moderately and poorly differentiated HCC. This result indicated that a dedifferentiation of HCC is associated with tumor proliferation.

This is a cross-sectional study and causal relationship between serum adiponectin level and dedifferentiation of HCC is unclear. One would think that dedifferentiation of HCC may trigger a mechanism which leads to decreased serum adiponectin level. However, it seems that hypoadiponectinemia may trigger dedifferentiation of HCC because of followings: Saxena et al. showed that adiponectin increased the phosphorylation of AMP-activated protein kinase (AMPK) and the TSC2 tumor suppressor, and inhibited phosphorylation of the mammalian target of rapamycin (mTOR) in vitro assay using Huh7 and HepG2 HCC cells [11]. Moreover, microarray analysis of tissue adiponectin expression levels in human HCC patients revealed that adiponectin expression was inversely correlated with tumor size [11]. Miyazaki et al. showed that adiponectin stimulated c-Jun NH2-terminal kinase (JNK) activation and suppressed signal transducer and activator of transcription 3 (STAT3) activation in HepG2 HCC cells [33]. Thus, these studies support that adiponectin level may inhibit proliferation and differentiation of HCC.

Angiogenesis is also an important process for proliferation, dedifferentiation, and metastasis of HCC. In small sized and well-differentiated HCCs, artery-like vessels are not well developed [34]. On the other hand, in moderately or poorly differentiated HCCs with 2 cm or larger, artery-like vessels are well developed [35], and these tumors display high proliferation and metastasis so-called advanced stage. Vascular endothelial growth factor (VEGF) is an endothelial cell-specific mitogen, and is the most important factor in tumor angiogenesis [36]. Yamaguchi et al. showed that VEGF expression in well-differentiated HCC was higher than expression in moderately and poorly differentiated HCC [37]. Well-differentiated HCCs that are 1.0–1.5 cm would be in the transitioning from the portal blood supply to an arterial blood supply, which would result in increased VEGF expression because of relative hypoxia from low blood flow. Therefore, high VEGF expression in small-sized and Well-differentiated HCCs suggests that VEGF plays an important role during relatively early angiogenesis stages in HCC. Previous studies have reported on the the molecular mechanisms involved in the negative association of adiponectin with tumor angiogenesis [38], [39]. Man et al. showed in an orthotopic liver tumor nude mouse model that adiponectin suppresses tumor growth through inhibition of tumor angiogenesis [39]. The molecular mechanism involves adiponectin downregulation of VEGF expression through inhibition of tumor-associated macrophages in tumor tissue. Moreover, the nude mice administrated adiponectin had significantly lower circulating VEGF levels than the control. This result suggests that adiponectin may inhibit dedifferentiation in well-differentiated HCC by inhibition of tumor angiogenesis-related VEGF.

Adiponectin exists mainly in three forms [12]. HMW adiponectin is thought to have higher biological activity than the other forms of adiponectin, especially in the liver [12], [13]. Several studies have reported that HMW adiponectin levels or the ratio of HMW to total adiponectin is inversely and more strongly associated with metabolic risk factors than total adiponectin levels [40], [41]. However, it remains unknown whether HMW adiponectin has more strongly potential actions on cancer pathophysiology than total adiponectin. In this study, we demonstrated that total and HMW adiponectin were independent risk factors for HCC histological grade. However, odds ratio of these factors were similar and serum total adiponectin levels were significantly associated with serum HMW adiponectin levels in this study (data not shown). Thus, one would think that impact of HMW adiponectin may be equal to total adiponectin for predicting HCC histological grade and testing for either total or HMW adiponectin levels may be as effective as testing both levels.

In conclusion, our data suggested that serum total and HMW adiponectin levels were predictors of liver fibrosis, but not prevalence of HCC in patients with chronic HCV infection. Moreover, we showed that low total and HMW adiponectin levels were independent risk factors for worse histological grades of HCC. Further study will be focused on the causal relationship between hypoadiponectinemia and dedifferentiation of HCC.
